# Graphene oxide decorated multi-frequency surface acoustic wave humidity sensor for hygienic applications

**DOI:** 10.1038/s41598-023-34099-7

**Published:** 2023-04-26

**Authors:** Soon In Jung, Il Ryu Jang, Chaehyun Ryu, Jeonhyeong Park, Aneeta Manjari Padhan, Hoe Joon Kim

**Affiliations:** 1 Department of Robotics and Mechatronics Engineering, Daegu Gyeongbuk Institute of Science and Technology (DGIST), Daegu, 42988 Korea; 2 Robotics and Mechatronics Research Center, DGIST, Daegu, 42988 Korea

**Keywords:** Engineering, Electrical and electronic engineering, Mechanical engineering

## Abstract

This work presents the single-chip integration of a multi-frequency surface acoustic wave resonator (SAWR) based humidity sensor. Graphene oxide (GO), a humidity-sensing material, is integrated onto a confined sensing area of SAWR via electrospray deposition (ESD). The ESD method allows ng-resolution deposition of GO, optimizing the amount of sensing material. The proposed sensor consists of SWARs at three different resonant frequencies (180, 200 and 250 MHz) with a shared common sensing region, thus allowing direct analysis of sensor performances at different operating frequencies. Our findings reveal that the resonant frequency of the sensor impacts both measurement sensitivity and stability. A higher operating frequency ensures better sensitivity but suffers from a larger damping effect from absorbed water molecules. The maximum measurement sensitivity of 17.4 ppm/RH% is achieved with low drift. In addition, the developed sensor exhibits improved stability and sensitivity by as much as 150% and 75% in frequency shift and Quality factor (*Q*), respectively, by carefully selecting the operating frequencies at a given RH% range. Finally, the sensors are used for various hygienic applications, such as non-contact proximity detection and face mask inspection.

## Introduction

The demand for humidity sensors with high reliability and measurement sensitivity is higher than ever as the sensor is used in environmental monitoring, medical diagnosis, and indoor air quality assessment^1–3^. Above all, monitoring human respiration and air quality is crucial for determining clinical diagnoses among humans and the possibility of respiratory pathogens’ atmospheric dispersion^4,5^. The key factors that need to be considered in humidity sensors are robustness, measurement accuracy, selectivity, low limit of detection (LOD), and fast response/recovery time. Furthermore, the compactness of the sensor and complementary metal-oxide semiconductor (CMOS) compatibility is required for practical applications.

A piezoelectric microelectromechanical systems (MEMS) resonator is a good candidate for satisfying the requirements mentioned above, and a surface acoustic wave resonator (SAWR) has drawn much interest due to its simple fabrication process, intuitive sensing mechanism, high sensitivity, and measurement stability^6,7^. AlN piezoelectric thin film can be sputter deposited, ensuring CMOS compatibility^8,9^. In addition, the high acoustic velocity^10^, low loss, and chemical stability^11^ of AlN contribute to the development of MHz-GHz range resonant frequency (*f*_0_) and stable device operation in humid conditions. In addition, as *f*_0_ depends on the pitch of electrodes, multi-frequency SAWRs can be achieved in a single wafer or chip^12^.

SAWR-based humidity sensors display outstanding sensitivity compared to macroscale resonators, such as a quartz crystal microbalance (QCM), because of their high operating frequency and small form factor. However, SWAR-based humidity sensors may become vulnerable to high relative humidity (RH) conditions as the viscous medium induces a significant amount of damping under fast-vibrational motions, which induces significant damping and hinders the stable sensor operation. The induced mechanical damping effect strongly affects the amount of acoustic energy stored in the resonator and reduces the device quality factor (*Q*), an important parameter that directly impacts the stability of the sensor and integrability of the resonator-embedded oscillators^13–15^. Therefore, the careful selection of operating frequency depending on RH should be considered.

To enhance the current state of SAWR-based humidity sensors, the single-chip integration of multi-frequency AlN-SAWRs, consisting of three pairs of interdigitated transducers (IDTs), reflectors, and a single metalized electrode on a common delay line (CDL), is proposed in this work. Placing the CDL at the center, between the three pairs of IDTs, contributes to forming standing waves and mass sensitivity, which comprise the active/sensing area of SAWR sensors. Such a single-chip multi-frequency SAWR system allows a detailed study of the relationship between the operating frequency and sensor performance.

The amount of sensing material must be carefully accounted for a reliable sensor operation. Previously reported SAWR-based humidity sensors to implement conventional coating methods of the water molecule adsorption layers, such as spin coating, drop casting, and dip coating^16,17^. Although these conventional coating methods facilitate the fast and simple formation of sensing layers, the sensing material amount and deposition area cannot be accurately controlled. Moreover, undesired agglomerations of sensing materials happen due to the coffee-ring effect, resulting in uneven adsorption of water molecules and ultimately affecting the device performance. The proposed electrospray deposition (ESD) method overcomes the limitations of traditional coating methods, such as spin-coating, dip-coating, and drop-casting^14^.

Previous studies have employed various sensing materials, such as polymers (PVA, PEI), carbon nanotubes, and metal oxide (TiO_2_). However, these materials often exhibit poor mechanical durability, such as swelling, wear, and peeling off. Moreover, the viscosity of the adsorbed water molecules at high RH values (> 85% RH) often causes a significant damping effect, resulting in unreliable sensing output and no resonant peak^18,19^. Such issues ultimately limit the RH sensing range and lifetime of the sensor. To overcome these challenges, researchers have recently focused on graphene oxide (GO), which exhibits exceptional mechanical properties. A GO possesses an extraordinary structure that includes abundant hydrophilic functional groups on hydrophobic six-membered carbon rings^20^. Therefore, while the hydrophilic group of GO can trap many water molecules under high RH conditions, the hydrophobic six-membered carbon rings provide rigid support for the GO structure, suppressing the accumulation of water molecules and exhibiting minimal viscous effects on the resonator^21^, as shown in Fig. S1.

Although GO is a promising humidity sensing material, previously reported coating or integration methods may degrade GO quality and sensor performance. In an effort to maintain GO quality and precisely control the amount and deposited area of the GO layer, an electrospray deposition (ESD) method is presented in this paper. The suggested method enables the generation of nano or micron-sized droplets owing to high electrical voltage. Therefore, the strong electrostatic repulsion between the charged GO droplets suppresses the agglomeration and coffee-ring effects. Furthermore, grounding a CDL during ESD results in the area-controlled and precise deposition of GO. Such an approach allows in-depth analysis of various sensing parameters (i.e. measurement sensitivity, *Q*, and noise) as functions of *f*_0_ and the amount of GO composite. To demonstrate possible hygienic application of the sensor, it is used for non-contact proximity detection and faces mask inspection.

## Materials and methods

### Fabrication of multi-frequency AlN-SAWR

AlN was chosen as the piezoelectric material in this study owing to its moderate deposition temperature (350 °C) and CMOS compatibility. A 1-μm-thick AlN layer was sputter deposited on a high-resistivity 6-inch Si wafer (> 10^4^ Ω∙cm). An X-ray diffractometer (Malvern Panalytical Empyrean, UK) was employed to validate the quality of the AlN layer. Gold electrodes, patterned on a deposited AlN layer via photolithography and lift-off, generated surface acoustic waves. The dimensions of the device are summarized in Table 1.

**Table 1:** Design parameters of the multi-frequency SAW resonators.

Parameter	Description	Value
*λ*	Acoustic wavelength (= Period of IDT)	20, 24, 28 μm
*W*_i_	Width of IDT	λ/4
*W*_r_	Width of reflector	λ/4
*L*_i_	Length of IDT aperture	33 λ
*L*_r_	Length of reflector	33 λ
*N*_i_	Number of IDT	60
*N*_r_	Number of reflectors	90
*A*_CDL_	Area of common delay line	1.8 mm^2^

The SAWR was constructed using three pairs of IDTs, a CDL, and reflectors. The distance between the electrodes on the IDTs and reflectors was one-quarter of the acoustic wavelength, which is a photo lithographically controllable factor. The dispersion relation, in which the acoustic wavelength determines the resonant frequency, was used to fabricate the SAWR with precisely predicted *f*_0_ values via photolithography. Three pairs of IDTs and reflectors were circularly placed to generate waves with three resonant frequencies in a single chip. This configuration resulted in a shared region between the IDTs, the sensing area. This region was defined using a common delay line (CDL). The gold layer covered the CDL to create an electrically short sensing area, thus reducing the sensing error caused by the change in electrical properties during the adsorption of water molecules. In addition, the grounded gold layer enabled the GO to be selectively deposited on the CDL region via the electrostatic effect during ESD.

### Preparation of GO solution and electro spray deposition

The purchased GO (Sigma Aldrich, USA) was dispersed in DI water to obtain a 2 mg/mL concentration. To efficiently break up the GO solution during ESD, ethanol solvent was added to the GO solution until a concentration of 146 µg/mL was achieved. Then, the GO solution was immersed in an ultrasonic bath for 1 h. A syringe pump (Harvard Apparatus PHD Ultra, USA) was used to push the GO solution at a constant flow rate. For electric field focusing, the needle-shaped stainless was employed as a nozzle. The injected GO solution was broken up into multiple droplets due to the high electric field at the end of the nozzle. For a stable cone-jet spraying mode, the SAWR was placed at a distance of 45 mm from the nozzle, and a voltage of 4.9 kV was applied to the GO solution. A hot plate (Misung Scientific, Korea) heated the SAWR to evaporate the droplets and rapidly suppress the fusion of droplets. The coated GO layer was characterized using a Raman spectrometer (Nanobase XperRAM, Korea), an XRD, an optical microscope, and a scanning electron microscope (SEM) (Hitachi S4800, Japan).

### Humidity sensing and human activity monitoring experiments

The relative humidity (RH) levels of saturated salt solutions depend on the type of salt used^22^. In this study, RH chambers were made using eight kinds of salts: LiCl, MgCl_2_, K_2_CO_3_, NaBr, CuCl_2_, NaCl, KCl, and K_2_SO_4_ (Samchum Chemicals, Korea), with 11%, 33%, 43%, 58%, 67%, 75%, 85% and 97% RH, respectively. The RH level of each chamber was verified using a digital hygrometer (Elitech MAXMIN-24, Korea). To avoid the effects of any previously adsorbed water molecules, the SAWR was dehumidified in an 11% RH chamber for 2 min and then exposed to each RH chamber for 2 min. A vector network analyzer (VNA; Agilent E5071C) was used to monitor the frequency response, such as the *f*_0_ and *Q,* of the sensor. All measurements are taken at atmospheric pressure and a temperature of 25 °C using ambient air as the base gas.

To test the SAWR’s applicability in human activity monitoring, humidity caused by moisture on a finger and respiration were measured. The SAWR was first made to track the humidity field generated by moisture on a finger. To demonstrate its motion-sensing performance, the device was tested with the moving speed of the finger and the distance between the finger and the sensor. A bare finger, a finger with moisturizing cream, and a finger covered by a glove were used for the moisture sensor analysis. Next, in the respiration monitoring test, the SAWR was expected to detect the level of moisture and saliva droplets at breathing flow. Furthermore, to demonstrate its ability to sense respiration rates, the SAWR was tested with a normal respiration rate (12–16 bpm) and a rapid respiration rate (40–60 bpm). Additionally, to evaluate the filtration efficiency of the face masks, respiration rates with the Korean filter 94 mask (KF 94; 94 denotes the filtration efficiency) and dental mask were tested.

## Results and discussion

**Figure 1:**
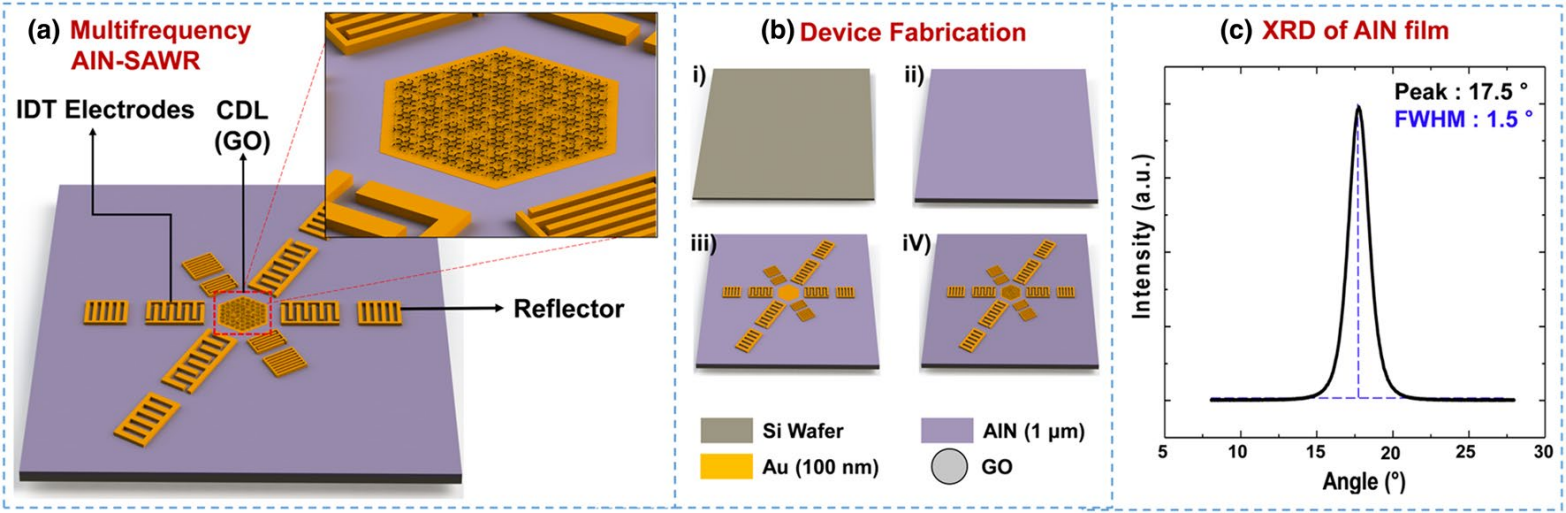
(**a**) The configuration of SAWR consisting of IDTs, reflectors, and CDL. (**b**) A fabrication process of SAWR with a GO layer. (**c**) XRD analysis of AlN layer.

Figure 1a shows the configuration of the multi-frequency SAWR, consisting of three pairs of IDTs, reflectors, and a CDL, developed in this study. The IDTs and reflectors were circularly placed on an AlN layer to generate surface acoustic waves with different resonant frequencies. In this configuration, all the operating patterns shared a sensing area, thus enabling resonant frequency-dependent analysis under the same amount of water molecule adsorption. Figure 1b depicts the fabrication process used for the SAWR humidity sensor. First, a 1-µm-thick AlN layer was sputtered on a high-resistivity 6-inch silicon wafer. Then, a 100-nm-thick Au electrode was patterned on the AlN layer via photolithography and lift-off process to make the electrode configuration of the SAWR. A substrate dicing system was used to cut the fabricated wafers into individual chips of 9.5 × 9.5 mm^2^. The printed circuit board (PCB) and wire bonder created electrical connections on the SAWR for the ESD of GO and measurement of electrical signals. Then, the GO film was selectively decorated on the CDL using the ESD. Here, the electrically grounded CDL attracted the charged GO droplets owing to the electrostatic effect, which resulted in selective deposition on the CDL.

The devices were characterized using XRD to investigate the crystalline structure of the AlN layer, as shown in Fig. 1c. The obtained major peak at 17.5° with full width at half maximum (FWHM) of 1.5° agrees with the AlN crystal orientation^23^. Table 1 shows the design parameters of the multi-frequency SAWR. The distances between the electrodes on both IDTs and reflectors were adjusted to generate acoustic wavelengths of 20, 24 and 28 µm, corresponding to *f*_0_ of 180, 200 and 250 MHz, respectively. To minimize wave loss when operating the SAWR, the design involved spacing the electrodes with an integer number of quarter wavelengths.

**Figure 2:**
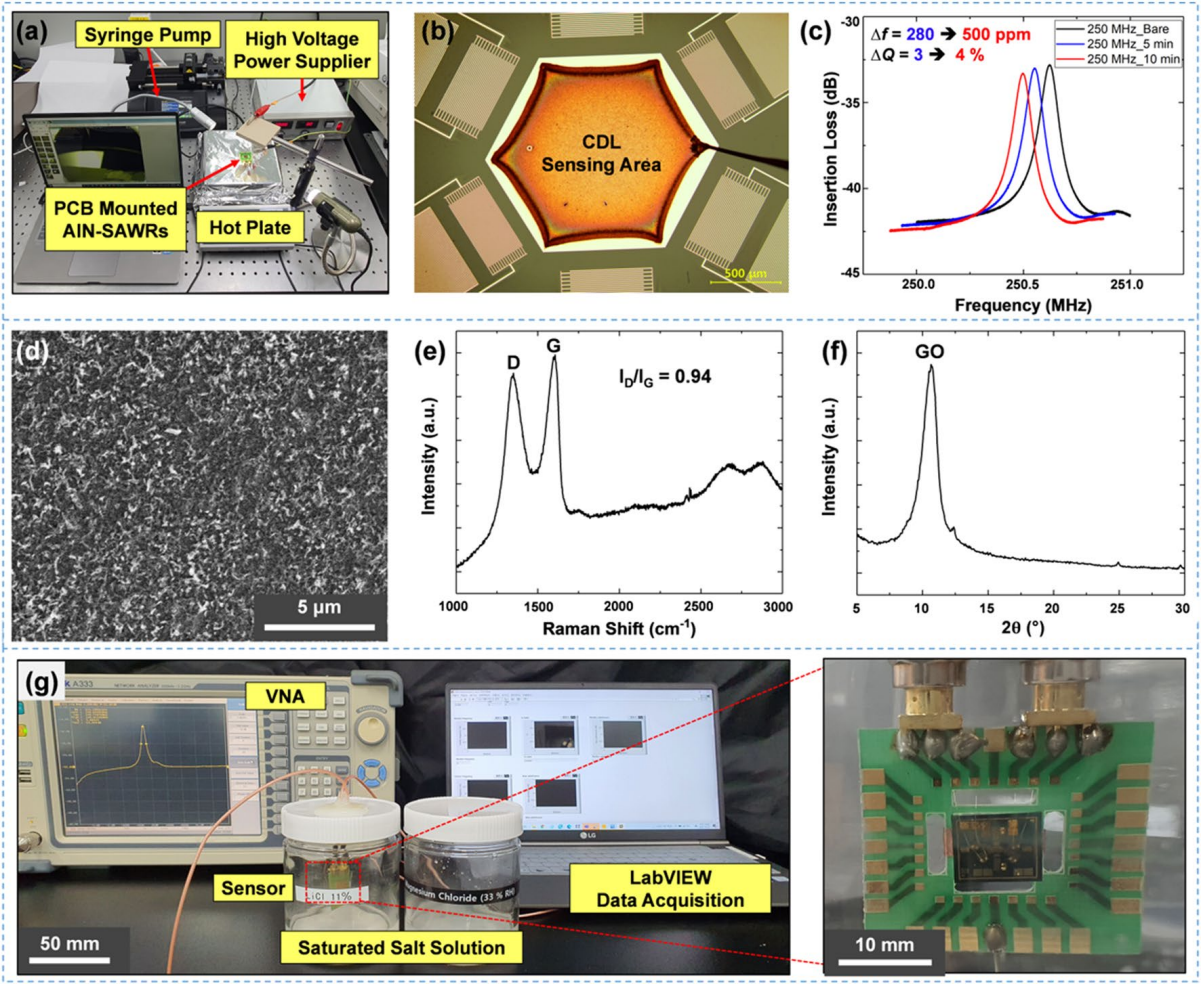
(**a**) The digital image of the ESD setup consisting of the syringe pump, high voltage supply, and hot plate. (**b**) The optical microscope image of coated GO film on CDL. (**c**) The resonant peak shift with GO spraying time for 250 MHz SAWR. (**d**) The scanning electron microscope (SEM) image of GO film. (**e**) Raman and (**f**) XRD analysis of coated GO film. (**f**) Experimental setup for testing SAWR humidity sensor consisting of VNA, LabVIEW connected PC, and saturated salt solution chamber. (**g**) The PCB mounted SAWR for an electrical connection.

Figure 2a shows the digital image of the ESD setup, consisting of the syringe pump, high voltage supply, and hot plate. The syringe pump was used to inject GO solution at a flow rate of 10 µL/min. The GO solution split into many droplets at the end of the nozzle, where a high electrical voltage was applied. The generated droplets traveled along the electric field line as they were polarized. Therefore, the GO film was coated only on the grounded CDL, as shown in Fig. 2b. The droplets rapidly evaporated as a hot plate heated the CDL at 80 °C.

Figure 2c shows the shift in the resonant peak with GO spraying time for the 250 MHz SAWR. The resonant frequency decreased with the amount of GO, while the Q reduction was less than 5%. This result shows that ESD is favorable for coating sensing materials on SAWR with minimal damping issues. When the GO droplets travel during ESD, the electrostatic repulsion alleviates the agglomeration of GO, leading to a well-dispersed GO film, as shown in Fig. 2d. Raman and XRD analyses were performed to characterize the GO film, as shown in Fig. 2e–f. The Raman spectrum exhibited D (1341 cm^−1^) and G (1596 cm^−1^) peaks. The intensity ratio of the D peak to the G peak was about 0.94, in agreement with past research^24^. The XRD data revealed a peak at 10°, corresponding to the (0 0 1) crystal plane of GO^25^. To validate the hydrophilicity of the GO-coated surface, we have measured the contact angle of water droplets on the Au electrode and GO-coated surface, as shown in Fig. S2.

Figure 2g shows the experimental setup for testing the SAWR humidity sensor consisting of a VNA, LabVIEW-connected PC, and saturated salt solution chamber. The wire bonding enabled an electrical connection between the SAWR and PCB, easing the connection of RF cable assemblies. A VNA measures the two-port S-parameter of a transmitted signal, while a LabVIEW-connected PC extracts *f*_0_ and *Q* in real-time. The SAWR was loaded into the RH chamber containing the saturated salt solution for 2 min to demonstrate humidity sensing performance. To minimize the sensing error caused by existing water molecules, the SAWR was dehumidified in the 11% RH chamber for 2 min before loading each chamber.

**Figure 3:**
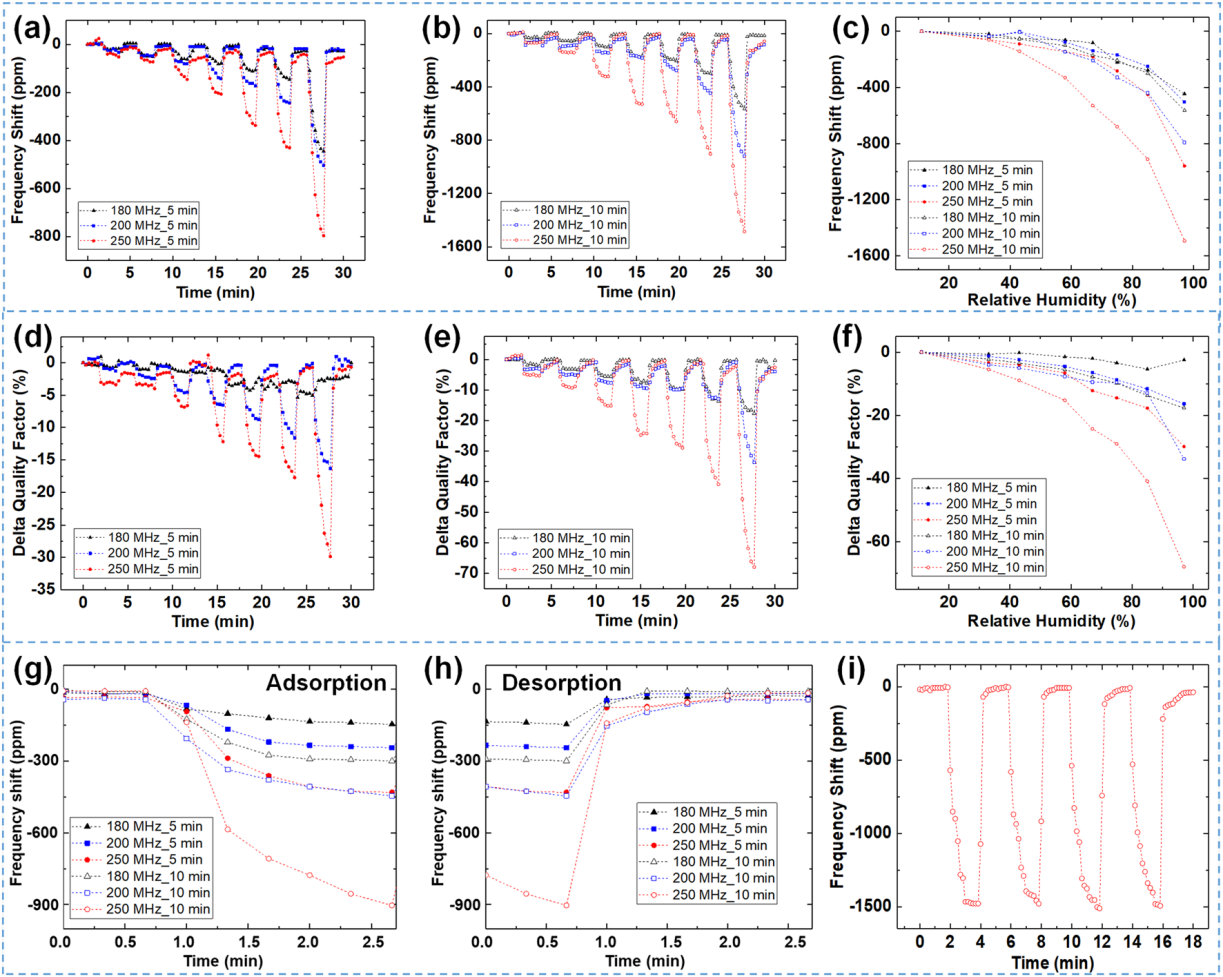
The frequency responses of the SAWRs with different GO masses under the various RH conditions. (**a,b**) The *f*_0_ shifts of the SAWR humidity sensors with different GO ESD times for 5 min and 10 min, respectively. (**c**) The shifts in *f*_0_ as a function of the deposited GO mass and the operating frequencies at different RH levels. (**d,e**) The device *Q* with 5 min and 10 min ESD time as a function of the exposed RH levels. (**f**) The device *Q* fluctuations of each SAWR sensor under the various RH levels. (**g,h**) The adsorption and desorption tests of the SAWR sensors under the 85% RH level. (**i**) The frequency repeatability test of 250 MHz_10 min sensor at 97% RH environment.

The humidity adsorption abilities of the SAWR sensors were investigated at RH levels ranging from 11 to 97%. Figure 3a,b shows the sensor *f*_0_ shifts with 2-min exposure to 33, 43, 58, 67, 75, 85 and 97% RH conditions and 2-min dehumidification at 11% RH. The results show that the sensor’s sensitivity strongly depends on the *f*_0_ of the SAWR sensors and the amount of GO layer. For example, at 97% RH, the 250 MHz_10 min device had twice the amount of GO compared to the 250 MHz_5 min device, resulting in about 1.6 times higher sensitivity. Thus, it can be concluded that the measurement sensitivity can be fine-tuned by adjusting the ESD time.

A higher operating frequency sensor is more sensitive to water molecule adsorption based on the Sauerbrey equation^26^. The relationship between the frequency shift (∆*f*) of the SAWR sensor and mass change (∆*m*) of the total system due to the water molecule adsorption into the GO layer could be analyzed using the following equation:

1 $${\Delta f}/{{f}_{0}}= -C{f}_{0}\Delta m/A,$$

where *C* is the constant, *f*_0_ is the fundamental frequency, and *A* is the sensing area (CDL). From the above equation, the frequency change in the SAWR sensor is a strong function of the *f*_0_ and mass added by the water molecule adsorption. Moreover, a larger amount of the GO layer contains more hydroxyl groups that induce an increase in the physiosorbed water molecules. Consequently, the total mass of the SAWR sensor shift under the presence of humidity. Among the SAWR-based humidity sensors, the 250 MHz_10 min (sensor with *f*_0_ of 250 MHz and 10 min of GO deposition) sensor exhibited the highest water molecule adsorption ability as it resulted in Δ*f* of 1500 ppm under the 97% RH condition, owing to its high *f*_0_ and a large amount of GO film.

The frequency responses of the SAWR-based humidity sensors under the various RH levels were monitored, as shown in Fig. 3c. All of the sensors showed nonlinear behaviors of the humidity sensing responses as the increase of the exposed RH conditions. This phenomenon was likely due to the increase in the distance between the interlayers of the GO film in the humid environment^27,28^. In other words, the water molecules penetrated the interlayers of the GO composite in high RH conditions, inducing nonuniform structures and separated layers of GO film, thus causing increased distances between the GO interlayers. The additional spaces in the GO composite allowed the water molecule adsorption capacity to increase, and then the frequency responses exhibited nonlinearity as a function of the RH levels.

The other key parameter of the resonant sensor is *Q*, which determines the device’s noise level and power budget. Figure 3d–f shows the *Q* data and exposed RH conditions in the present study. The *Q*s exhibit a similar trend in frequency responses. The higher *f*_0_ and larger amount of GO decorations of the sensor would be severely affected by the mass loading effect due to the adsorption of water molecules by the GO composite. Notably, although the 250 MHz_10 min sensor was found to have the highest sensitivity, it also provoked a *Q* reduction of 70% under the 97% RH condition due to the accumulation of water molecules and the high resonant frequency, resulting in substantial damping. Such a severe *Q* reduction would not be suitable for the practical utilization of the sensor in a humid environment.

Figure 3g,h shows the response and recovery performance of the SAWR sensors as the RH levels changed from 11 to 85%. For comparison with previously reported humidity sensors, the response and recovery times were defined as the time taken to reach 90% of the final value. The measured response times ranged from 54 to 68 s, and the recovery times ranged from 12 to 22 s, comparable to the previously reported values for SAW-based humidity sensors^29,30^. Figure 3i shows the repeatability of 250 MHz_10 min sensor with 4 cycles test under 97% RH level. The SAWR sensor exhibits good repeatability without severe frequency drift during the test.

We measured the resonant frequencies and calculated the noise level of 180 MHz, 200 MHz and 250 MHz devices for 2 min under 11% RH conditions, as shown in Fig. S3. Due to the vulnerability of high resonant frequency SAW devices to air damping, the 250 MHz device exhibited the highest noise level with an *f*_STDEV_ of 2.585 kHz (10.5 ppm). The limit of detection (LOD) was then calculated using the following equation^31^:

2 $$\mathrm{LOD }= \left(3\times \frac{{f}_{STDEV}}{Sensitivity}\right) [\mathrm{\% RH}]$$

The resulting LOD values for 180 MHz, 200 MHz, and 250 MHz were 2.4%, 2.7% and 1.8% RH, respectively.

The device *Q* directly impacts the sensor performance and the compatibility of RF components (i.e. oscillator, antenna, filters) with resonator-based sensors. Specifically, a lower *Q* induces unnecessary bandwidth operations in the sensor and resonator-embedded oscillation systems. Thus, the highest *Q* had to be maintained to improve a device’s noise level and ensure stable oscillator operation^32,33^. To improve the reduction of the device *Q* in a humid environment, the optimal operating frequency was selected to ensure a stable measurement sensitivity while maintaining a high *Q*.

**Figure 4:**
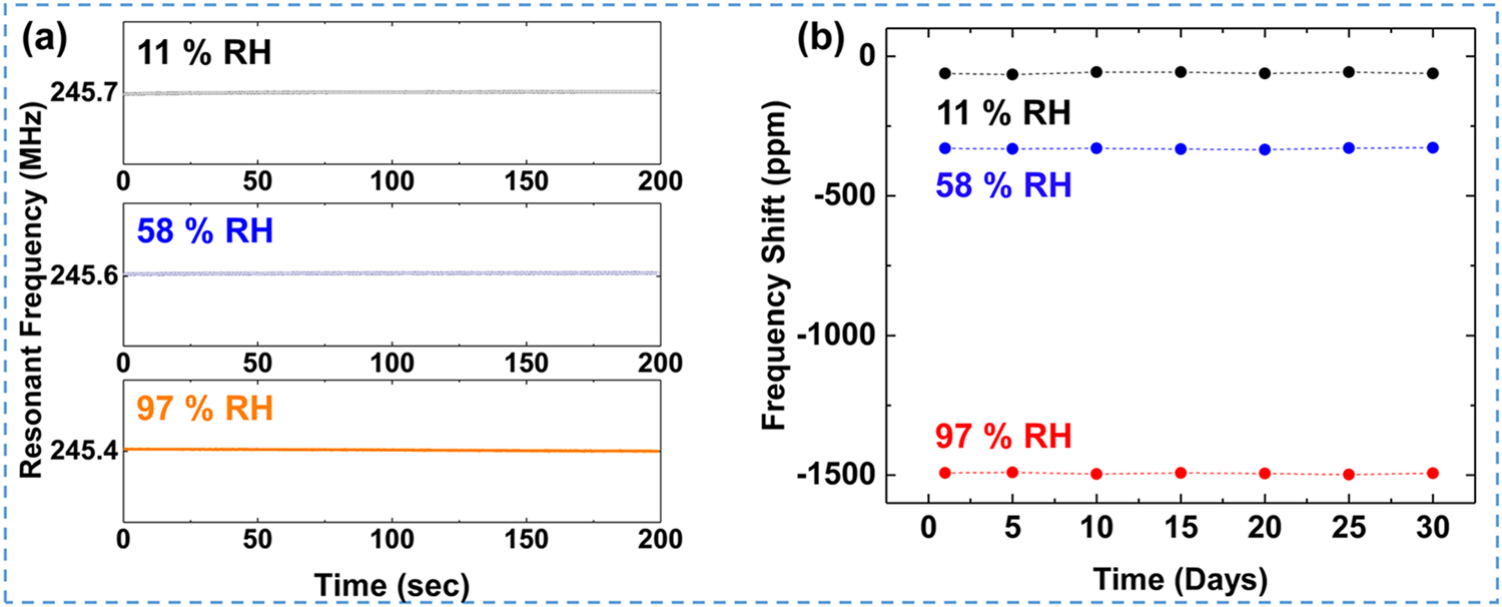
(**a**) The short-term stability test of 250 MHz_10 min sensor under the different RH levels. (**b**) The long-term stability test of the SAWR sensor for 30 days of operation.

Figure 4a shows the *f*_0_ of the 250 MHz_10 min sensor at various RH levels of 11%, 58%, and 97%. The sensor exhibited excellent stability overall at each RH level, with small variations in the *f*_0_ less than ± 10 ppm shift. The long-term stabilities under different RH levels are investigated, as shown in Fig. 4b. Our device guarantees the desired frequency stabilities with just a few ppm scale drifts, even under the highest RH conditions.

**Figure 5:**
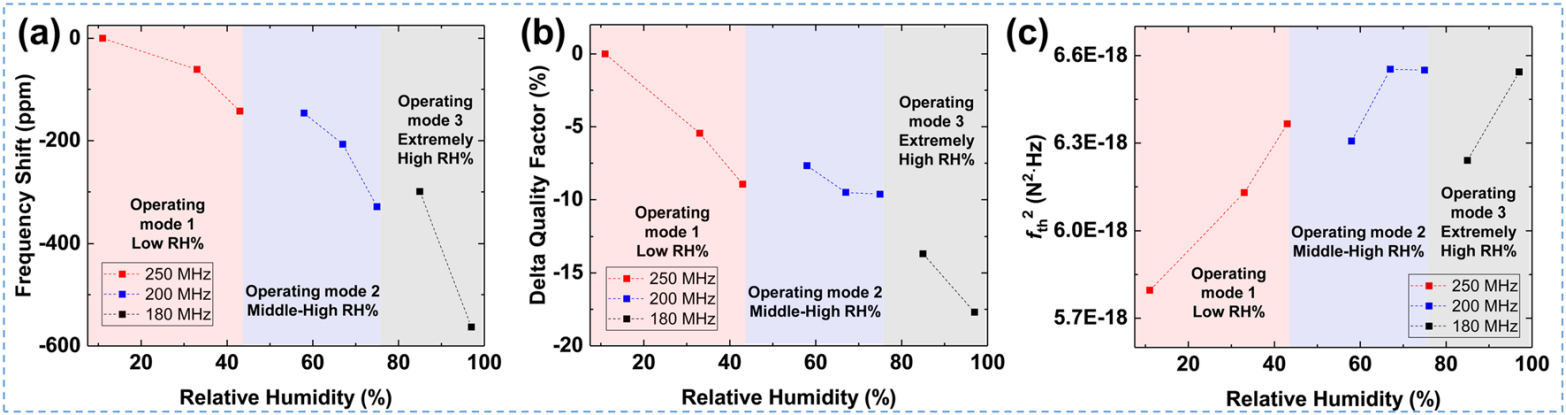
The resonance characteristics and thermal noise force responses by selecting the operating frequency according to the exposed RH conditions. (**a,b**) The frequency shifts and *Q* responses of the selected SAWR as the function of RH conditions. (**c**) Thermal noise forces along with the selected SAWR under the various RH conditions.

Figure 5a,b exhibits the *Q* responses with different operating frequencies as a function of the exposed RH levels. The results show that for lower RH levels, the sensor with the highest humidity sensitivity, i.e. the 250 MHz SAWR sensor, is preferable as it can trap many water molecules compared to other sensors. For the mid-range humidity conditions, specifically from 50 to 76% RH, the 200 MHz SAWR sensor is a good candidate as it provides moderate sensitivity and an improved degradation of *Q* value at about 10% under the 76% RH condition. Notably, the 180 MHz SAWR sensor provides the most improved degradation of *Q* value at less than 20% under the extremely humid environment above 80% RH; thus, it enables the original resonance characteristics to be maintained even while loading large amounts of water molecules. Therefore, the aforementioned approaches can be used for wide-range detection in various humidity levels while sustaining the device’s high *Q*.

With MEMS resonators, the noise processes act on the short-term stability of the resonators and randomly vary the operating frequencies^34^. The minimized device form factor and high operating frequencies prohibit the optimal resonance effects compared to macroscale resonators, such as QCM^35^. One of the most significant noise processes is thermal noise, which is the most fundamental fluctuation of any resonator, along with the overall operating frequency^34,36^. The equation below describes the relation between thermal noise forces (*f*_th_^2^) and the resonance factors of a resonator^37^.

3 $${{f}_{\mathrm{th}}}^{2}=4{k}_{\mathrm{B}}Tm{\omega }_{0}/Q$$

where *k*_B_ is the Boltzmann constant, *T* is the temperature, *ω*_0_ is the angular resonant frequency, *m* is the effective mass of the resonator, and *Q* is the quality factor. According to the above equation, the thermal noise force is most relevant to the device’s *Q*. Additionally, the thermal noise effect is proportional to the phase noise in resonator-combined oscillator systems^37,38^. Thus, it is clear that *Q* should be considered to ensure the sensor’s stable operation and integrability with CMOS oscillators. Figure 5c depicts the thermal noise force responses of the SAWR sensors as a function of the exposed RH conditions. Among the SAWR-based humidity sensors, the 180 MHz SAWR sensor exhibited the best thermal noise (below 6.6 × 10^−18^ N^2^∙Hz) performance even under humid conditions because it was less affected by the mass loading of the water molecules. The device’s Q could be maintained under the overall humid environment through the suggested system, allowing for the stable operation of the resonator-based sensors and oscillator block systems.

Table 2 summarizes the device’s main parameters (i.e. sensitivity, *Q*s, and thermal noise) of 180 MHz and 250 MHz sensors under 33% and 97% RH levels. For lower RH conditions of 33%, the 250 MHz_10 min sensor guarantees 150% higher measurement sensitivity compared to the 180 MHz sensor with an insignificant reduction of the device *Q*. In the case of humid conditions of 97% RH, the 180 MHz sensor provides an outstanding improvement in device *Q* compared to the 250 MHz sensor with moderate sensitivity. The reduction of the device *Q* is only about 26% compared to that of a 250 MHZ sensor. Although the lower *f*_0_ devices cannot provide enormous sensitivity, it ensures high *Q* even under extremely humid environments, ensuring a stable sensor operation in highly humid conditions. In other words, the lower *f*_0_ sensor is less affected by the mass loading effect (water molecules adsorption).

**Table 2:** Comparison of device key parameters of 180 MHz_10 min and 250 MHz_10 min sensors under 33 and 97% RH conditions.

Parameter	RH: 33%		RH: 97%	
	180 MHz 10 min	250 MHz 10 min	180 MHz 10 min	250 MHz 10 min
Δ*f* (ppm)	− 41.6	− 61.1	− 563.4	− 1493
Delta *Q*-factor (%)	− 2.3	− 5.4	− 17.7	− 67.9
Thermal noise (N^2^∙Hz∙10^−18^)	5.9	6.1	6.5	7.4

**Figure 6:**
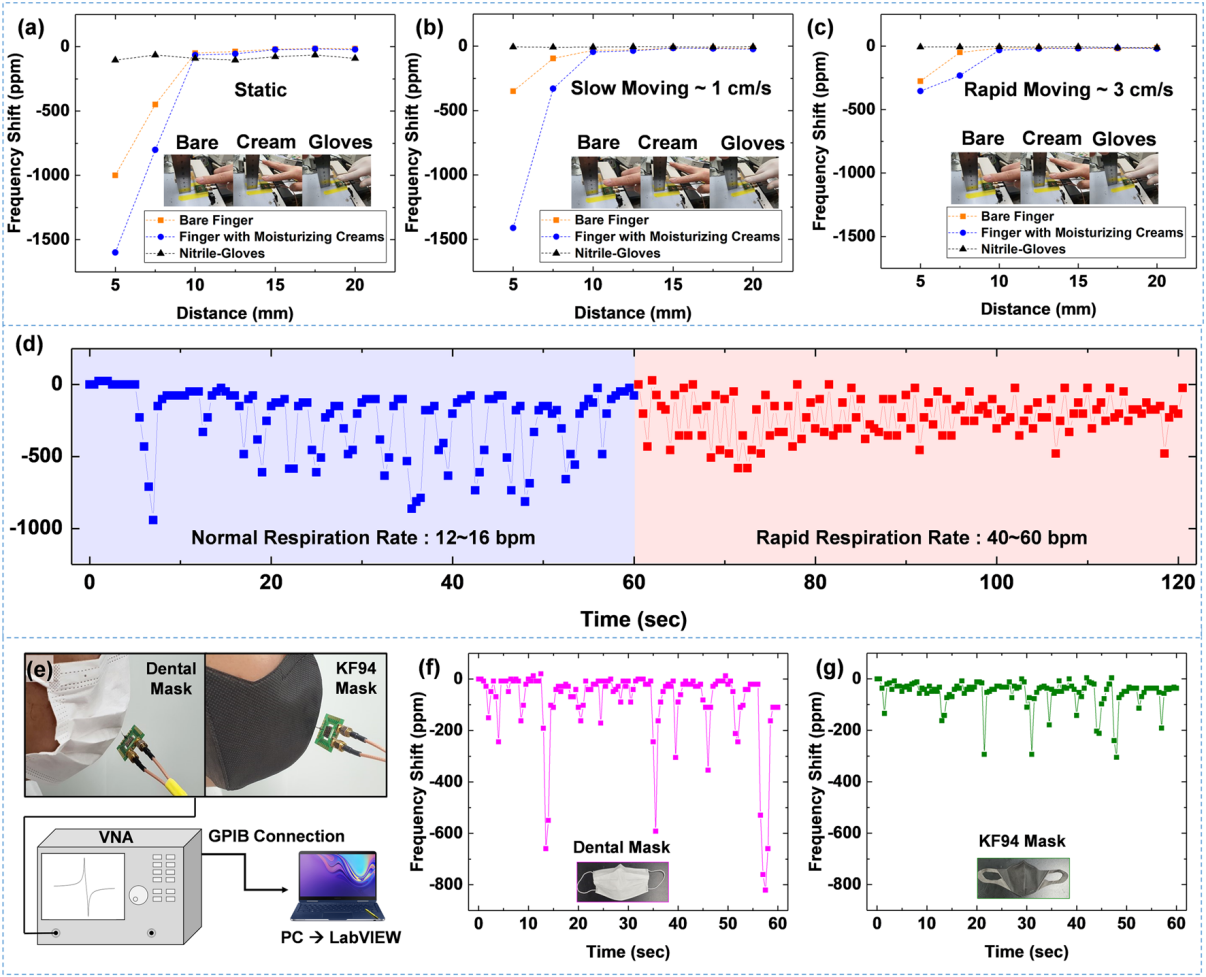
The frequency responses of the SAW 250 MHz_10 min sensor at different distances between the finger and sensor. The three kinds of the finger (i.e. bare finger, finger with moisturizing creams, and finger with nitrile gloves) are prepared. The sensors are tested at different speeds of finger motion, which are (**a**) static, (**b**) slow-moving (1 cm/s), and (**c**) rapid-moving (3 cm/s). (**d**) The monitoring of respiration rates using SAW 250 MHz_10 min sensor. (**e**) The schematic diagram of the experimental setup for testing salvia droplet filtration efficacy with face masks (dental mask and KF 94 mask). The frequency response of SAW 250 MHz_10 min sensor for the respiration with (**f**) dental mask and (**g**) KF 94 mask.

Water moisture level on human skin provides valuable information on human health and skin conditions. In addition, highly sensitive humidity sensors can detect proximity and contact for human–machine interfaces and hygienic applications. The sensors developed in the present study are applied to the rapid sensing of humidity generated from human skin, as shown in Fig. 6a–c. Moisture on the human finger generates a millimeter-scaled humidity field. To quantify this humidity field, the sensor-to-finger distance, amount of moisture on the finger (bare, lotion cream applied, and gloves on), and moving speed of the finger were chosen as experimental variables. The 250 MHz_10 min sensor was employed for this experiment. The finger with nitrile glove showed a slight frequency shift due to dry skin condition.

In contrast, the sensor accurately detected a humidity field at a 10 mm distance in the tests involving a bare and moisturized finger. Specifically, the moisturized finger caused the largest shift in resonant frequency. This result indicates that the developed sensor could detect humidity fields with moisture content on human skin, indicating its potential application in human healthcare monitoring and proximity detection. Furthermore, the sensor showed different outputs as the speed of finger motion changed: static, slow-moving (1 cm/s), and rapid-moving (3 cm/s). Thus, the sensor can detect finger position, moisture level, and finger motion. The humidity sensing performance in this study evidences that GO-coated SAWR can be used to develop a non-contact switch system and proximity detection.

Human respiration monitoring is widely used to analyze human activity and diagnose respiratory disorders. Moreover, the tiny saliva droplets generated from respiration could serve as carriers for the transmission of viruses. The respiration rate sensing performance of the 250 MHz_10 min sensor was tested in this study. The sensor was exposed to breath flow for 60 s in normal respiration (12–16 bpm) and rapid respiration (40–60 bpm) conditions. Figure 6d shows that the sensor accurately detected respiration rates with excellent sensitivity. This result indicates that the sensor developed in this study can be used in the human healthcare monitoring system. Further, the sensor was used to test the saliva droplet filtration efficiency of the dental mask and KF 94 mask, as shown in Fig. 6e. Respiration with KF 94 induced a lower frequency shift than respiration with the dental mask, indicating its higher filtering efficiency of saliva droplets, as seen in Fig. 6f,g. It was also found that the GO-coated SAWR can quantify the saliva droplets. There are still many challenges in detecting airborne viruses, including developing a target virus-capturing system and sampling airborne viruses. However, we believe this study could potentially contribute to developing a detecting system for analyzing aerosol transmission.

Table 3 compares the performance of the developed sensor with reported resonator-based humidity sensors^39–45^. While the cantilever-type devices have demonstrated higher sensitivity than the presented devices, their performance is limited at high RH levels (> 60% RH) due to excessive loading of water molecules and unstable device operation. Furthermore, although the optical thermally actuated resonator shows superior sensitivity across the overall range of RH, this sensor is likely to become unreliable due to significant temperature-dependent reswponse and requires an additional optical detecting system, such as a lock-in amplifier and photodetector. In contrast, the presented SAWR device (250 MHz_10 min) exhibits comparable or even better measurement sensitivity of 17.4 ppm/% RH over a wider range of RH levels than previous studies, owing to the efficient integration of sensing materials using ESD and selective operation of resonant frequencies depending on RH levels.

**Table 3:** Comparison of humidity sensing performance between this work and previously reported work.

Device	Resonant frequency [MHz]	Humidity range [%]	Sensing material	Sensitivity [ppm/% RH]	Ref
BAW	10	30–75	PAN/PEI	16.4	^39^
BAW	6	11.3–97.3	Chitin nanofiber	9.8	^40^
BAW	10	11–95	Graphene quantum dots chitosan	3.9	^41^
Resonant microcantilever	0.178	30–60	ZnO	1556	^42^
Electrothermal actuator	0.017	30–80	ZnO nanorod	4.4	^43^
FBAR	1247	3–83	GO	5.3	^44^
Optical thermally actuated resonator	0.375	0–100	Graphene	933	^45^
*SAW*	*250*	*11–97*	*GO*	*17.4*	*This work*

Significant values are in italics.

## Conclusion

This paper presented the single-chip integration process of multi-frequency AlN-SAWRs, consisting of three pairs of IDTs with different *f*_0_ and a single CDL as a common sensing area. In addition, ESD was introduced in the sensing layer formation method, surpassing the traditional coating methods of spin coating, drop casting, and dip coating. The deposited GO composite exhibited a uniform structure and selective area formation on the CDL after ESD. Through the suggested integration, the impact of the amount of GO on sensor performance was analyzed. Although sensors with higher *f*_0_ and larger amounts of GO showed outstanding sensitivity, they also provoked a severe *Q* reduction in highly humid conditions. To address this limitation, operating frequencies were carefully selected depending on the exposed RH levels. The sensors maintained high *Q* and minimized the thermal noise effect in the various RH levels. In addition, the sensors could accurately measure the humidity fields on fingers and the saliva droplets resulting from respiration, enabling a promising sensor system for human healthcare monitoring and hygienic applications.

## Supplementary Information

Supplementary Figures.

## Data Availability

The datasets used and/or analyzed during the current study are available from the corresponding author on reasonable request.

## References

[1] Wu Z (2021). An excellent impedance-type humidity sensor based on halide perovskite CsPbBr 3 nanoparticles for human respiration monitoring. Sens. Actuators B Chem..

[2] Wang Y, Zhang L, Zhang Z, Sun P, Chen H (2020). High-sensitivity wearable and flexible humidity sensor based on graphene oxide/non-woven fabric for respiration monitoring. Langmuir.

[3] Lan L (2020). One-step and large-scale fabrication of flexible and wearable humidity sensor based on laser-induced graphene for real-time tracking of plant transpiration at bio-interface. Biosens. Bioelectron..

[4] Wang J (2021). Impact of temperature and relative humidity on the transmission of COVID-19: A modelling study in China and the United States. BMJ Open.

[5] He Z (2021). The influence of average temperature and relative humidity on new cases of COVID-19: Time-series analysis. JMIR Public Health Surveill..

[6] Guo Y (2022). A new strategy to minimize humidity influences on acoustic wave ultraviolet sensors using ZnO nanowires wrapped with hydrophobic silica nanoparticles. Microsyst. Nanoeng..

[7] Asadi K, Yeom J, Cho H (2021). Strong internal resonance in a nonlinear, asymmetric microbeam resonator. Microsyst. Nanoeng..

[8] Aslam MZ (2020). Surface acoustic wave modes characteristics of CMOS compatible SiO2/AlN/SiO2/Si multilayer structure with embedded electrodes. Sens. Actuators A.

[9] Ali WR, Prasad M (2020). Piezoelectric MEMS based acoustic sensors: A review. Sens. Actuators A.

[10] Lin CM, Chen YY, Felmetsger VV, Senesky DG, Pisano AP (2012). AlN/3C–SiC composite plate enabling high-frequency and high-Q micromechanical resonators. Adv. Mater..

[11] Xiong S (2020). Stability studies of ZnO and AlN thin film acoustic wave devices in acid and alkali harsh environments. RSC Adv..

[12] Zhou J (2022). Record-breaking frequency of 44 GHz based on the higher order mode of surface acoustic waves with LiNbO3/SiO2/SiC heterostructures. Engineering.

[13] Kim HJ, Jung SI, Segovia-Fernandez J, Piazza G (2018). The impact of electrode materials on 1/f noise in piezoelectric AlN contour mode resonators. AIP Adv..

[14] Jang IR (2021). Direct and controlled device integration of graphene oxide on Quartz Crystal Microbalance via electrospray deposition for stable humidity sensing. Ceram. Int..

[15] Gao F, Bermak A, Benchabane S, Robert L, Khelif A (2021). Acoustic radiation-free surface phononic crystal resonator for in-liquid low-noise gravimetric detection. Microsyst. Nanoeng..

[16] Penza M, Cassano G (2000). Relative humidity sensing by PVA-coated dual resonator SAW oscillator. Sens. Actuators B Chem..

[17] Lu L (2019). High performance SnO 2/MoS 2-based surface acoustic wave humidity sensor with good linearity. IEEE Sens. J..

[18] Dong Y, Feng G (1995). Effects of surface physical sorption on characteristic of coated quartz-crystal humidity sensor. Sens. Actuators B Chem..

[19] Yao Y, Chen X, Li X, Chen X, Li N (2014). Investigation of the stability of QCM humidity sensor using graphene oxide as sensing films. Sens. Actuators B Chem..

[20] Mkhoyan KA (2009). Atomic and electronic structure of graphene-oxide. Nano Lett..

[21] Zhang D (2018). Facile fabrication of high-performance QCM humidity sensor based on layer-by-layer self-assembled polyaniline/graphene oxide nanocomposite film. Sens. Actuators B Chem..

[22] Cao Y, Chen Y, Mu T (2013). A new way to tune relative humidity: By saturated ionic liquid aqueous solutions. New J. Chem..

[23] Kamohara T, Akiyama M, Ueno N, Kuwano N (2008). Improvement in crystal orientation of AlN thin films prepared on Mo electrodes using AlN interlayers. Ceram. Int..

[24] Claramunt S (2015). The importance of interbands on the interpretation of the Raman spectrum of graphene oxide. J. Phys. Chem. C.

[25] Krishnamoorthy K, Veerapandian M, Yun K, Kim S-J (2013). The chemical and structural analysis of graphene oxide with different degrees of oxidation. Carbon.

[26] Bodenhöfer K, Hierlemann A, Noetzel G, Weimar U, Göpel W (1996). Performances of mass-sensitive devices for gas sensing: Thickness shear mode and surface acoustic wave transducers. Anal. Chem..

[27] Medhekar NV, Ramasubramaniam A, Ruoff RS, Shenoy VB (2010). Hydrogen bond networks in graphene oxide composite paper: Structure and mechanical properties. ACS Nano.

[28] Wu J (2020). Ultrathin glass-based flexible, transparent, and ultrasensitive surface acoustic wave humidity sensor with ZnO nanowires and graphene quantum dots. ACS Appl. Mater. Interfaces.

[29] Xu Z, Li Z (2021). Dynamic humidity response of surface acoustic wave sensors based on zinc oxide nanoparticles sensitive film. Appl. Phys. A.

[30] Yan X (2021). Surface acoustic wave relative humidity sensor based on sputtering SiO2 film. Surf. Interface Anal..

[31] Li J (2003). Carbon nanotube sensors for gas and organic vapor detection. Nano Lett..

[32] Jung SI, Ryu C, Piazza G, Kim HJ (2019). A study on the effects of bottom electrode designs on aluminum nitride contour-mode resonators. Micromachines.

[33] Jen H-T, Pillai G, Liu S-I, Li S-S (2020). High-Q support transducer MEMS resonators enabled low-phase-noise oscillators. IEEE Trans. Ultrason. Ferroelectr. Freq. Control.

[34] Walls FL, Vig JR (1995). Fundamental limits on the frequency stabilities of crystal oscillators. IEEE Trans. Ultrason. Ferroelectr. Freq. Control.

[35] Cleland AN, Roukes ML (2002). Noise processes in nanomechanical resonators. J. Appl. Phys..

[36] Vig JR, Kim Y (1999). Noise in microelectromechanical system resonators. IEEE Trans. Ultrason. Ferroelectr. Freq. Control.

[37] Miller JML (2018). Effective quality factor tuning mechanisms in micromechanical resonators. Appl. Phys. Rev..

[38] Cleland AN, Roukes ML (1996). Fabrication of high frequency nanometer scale mechanical resonators from bulk Si crystals. Appl. Phys. Lett..

[39] Rianjanu A (2020). Quartz crystal microbalance humidity sensors integrated with hydrophilic polyethyleneimine-grafted polyacrylonitrile nanofibers. Sens. Actuators B Chem..

[40] Chen Q, Liu D, Huang X-H, Yao Y, Mao K-L (2022). Impedance analysis of chitin nanofibers integrated bulk acoustic wave humidity sensor with asymmetric electrode configuration. Nanomaterials.

[41] Qi P (2019). A QCM humidity sensor constructed by graphene quantum dots and chitosan composites. Sens. Actuators A.

[42] Mistry K (2022). Highly sensitive self-actuated zinc oxide resonant microcantilever humidity sensor. Nano Lett..

[43] Xu J, Bertke M, Wasisto HS, Peiner E (2019). Piezoresistive microcantilevers for humidity sensing. J. Micromech. Microeng..

[44] Xuan W (2017). A film bulk acoustic resonator oscillator based humidity sensor with graphene oxide as the sensitive layer. J. Micromech. Microeng..

[45] Xiao X, Li C, Fan S-C, Liu Y-J, Liu Y (2023). Optical-thermally actuated graphene mechanical resonator for humidity sensing. Sens. Actuators B Chem..

