# Supplementary material for: Graphene oxide decorated multi-frequency surface acoustic wave humidity sensor for hygienic applications
Source: Sci Rep. 2023 Apr 26;13:6838. doi: 10.1038/s41598-023-34099-7 (PMC10133308; doi:10.1038/s41598-023-34099-7)
Supplement: Supplementary file 1 — Supplementary Figures. [file 41598_2023_34099_MOESM1_ESM.docx]

***Supplementary Information for***

**Graphene oxide decorated multi-frequency surface acoustic wave humidity sensor for hygienic applications**

*^^^****Soon In Jung^1^,*** *^^^****Il Ryu Jang^1^, Chaehyun Ryu^1^, Jeonhyeong Park^1^, Aneeta Manjari Padhan^1^, and *Hoe Joon Kim^1,2^***

^1^Department of Robotics and Mechatronics Engineering, Daegu Gyeongbuk Institute of Science & Technology (DGIST), Daegu 42988, Korea

^2^Robotics and Mechatronics Research Center, DGIST, Daegu 42988, Korea

**^** Authors with an equal contribution

***** Corresponding Author: Prof. Hoe Joon Kim (joonkim@dgist.ac.kr)


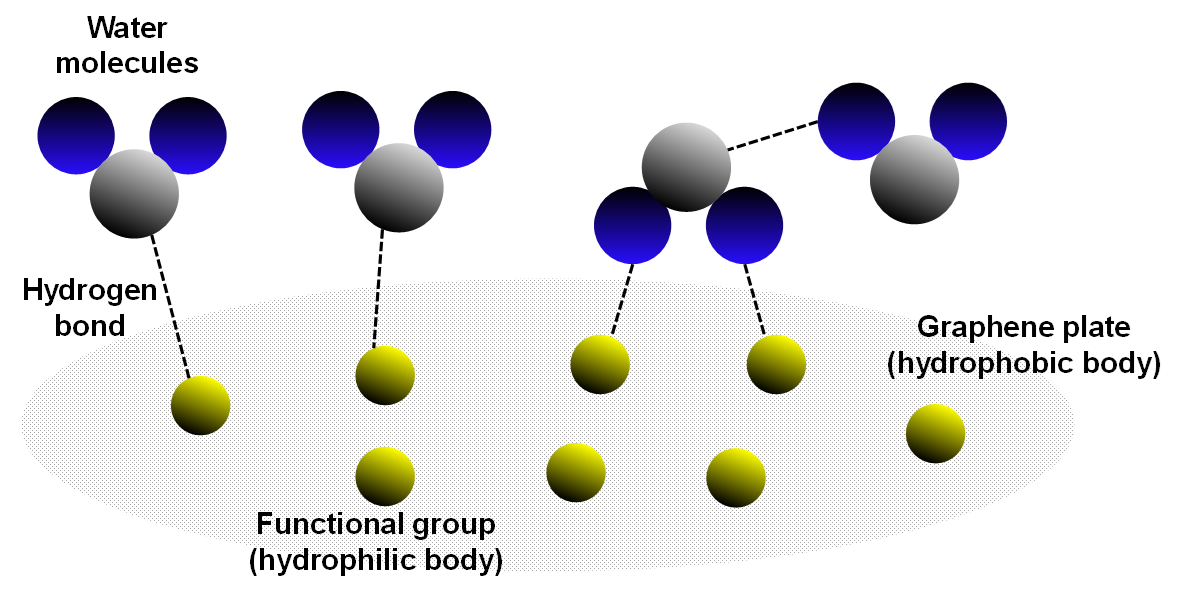


**Figure S1.** Humidity sensing mechanism of graphene oxide.


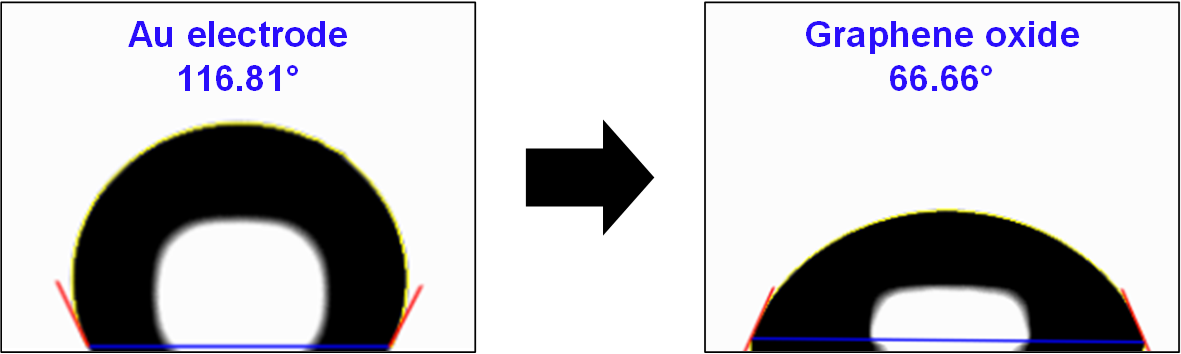


**Figure S2.** The contact angle of water droplets on both the Au electrode and GO coated surfaces.


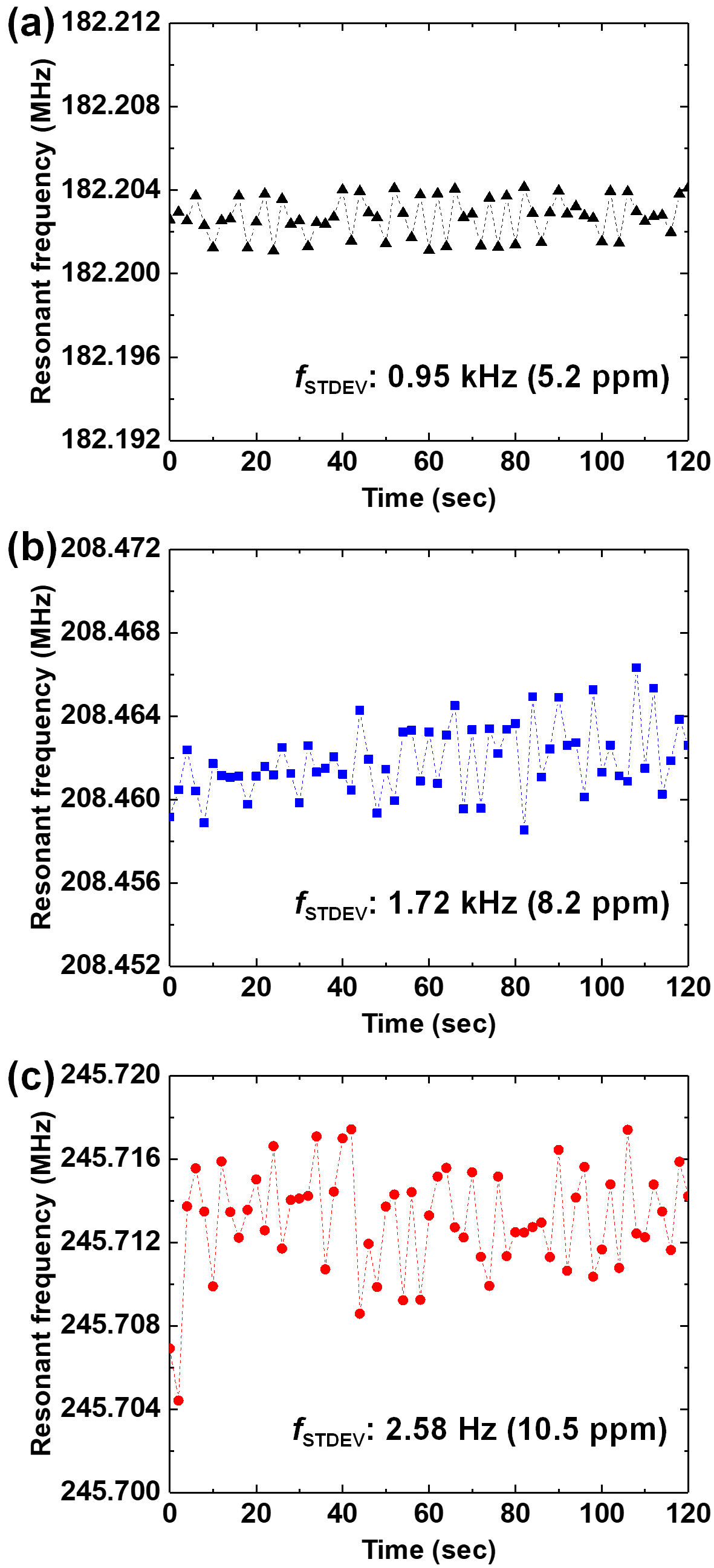


**Figure S3.** The resonant frquency and noise level for (a) 180 MHz, (b) 200 MHz, and (c) 250 MHz devices under 11% RH chamber.
